# Chromatin Remodeler CHD8 in Autism and Brain Development

**DOI:** 10.3390/jcm10020366

**Published:** 2021-01-19

**Authors:** Anke Hoffmann, Dietmar Spengler

**Affiliations:** Department of Translational Research in Psychiatry, Max-Planck Institute of Psychiatry, 80804 Munich, Germany; hoffmann@psych.mpg.de

**Keywords:** autism, neurodevelopmental disorders, chromatin regulation, neurodevelopment, neuronal connectivity, neurotransmission, neuronal plasticity, pluripotent stem cells

## Abstract

Chromodomain Helicase DNA-binding 8 (*CHD8*) is a high confidence risk factor for autism spectrum disorders (ASDs) and the genetic cause of a distinct neurodevelopmental syndrome with the core symptoms of autism, macrocephaly, and facial dysmorphism. The role of *CHD8* is well-characterized at the structural, biochemical, and transcriptional level. By contrast, much less is understood regarding how mutations in *CHD8* underpin altered brain function and mental disease. Studies on various model organisms have been proven critical to tackle this challenge. Here, we scrutinize recent advances in this field with a focus on phenotypes in transgenic animal models and highlight key findings on neurodevelopment, neuronal connectivity, neurotransmission, synaptic and homeostatic plasticity, and habituation. Against this backdrop, we further discuss how to improve future animal studies, both in terms of technical issues and with respect to the sex-specific effects of *Chd8* mutations for neuronal and higher-systems level function. We also consider outstanding questions in the field including ‘humanized’ mice models, therapeutic interventions, and how the use of pluripotent stem cell-derived cerebral organoids might help to address differences in neurodevelopment trajectories between model organisms and humans.

## 1. Introduction

The nuclear DNA of eukaryotic cells is tightly packaged with the help of histone proteins to form the so-called chromatin. This structure can adopt an open (transcriptionally active) or closed (transcriptionally inactive) configuration that critically controls the access of transcription factors (TFs) to their binding sites. Epigenetic factors control these chromatin states by imposing posttranslational modifications of free histone tails and by chromatin remodeling (reviewed in [1]). Chromatin states additionally depend on epigenetic factors imposing DNA modifications (e.g., DNA methylation) (reviewed in [2]). Both processes, modification of chromatin and of DNA, closely interact with each other and play an important role in the precise temporal and spatial control of gene expression during development and beyond. Consistent with this scenario, epigenetic factors have been increasingly recognized for their role in the initiation and progression of mental diseases (reviewed in [3,4]). 

Mutations in genes that encode for chromatin remodelers (CRs) have been originally identified as the genetic cause of distinct neurodevelopmental syndromes (reviewed in [5,6]). In addition, genome-wide association studies (GWAS) on neurodevelopmental disorders, including intellectual disability (ID) and ASD, have reported genetic variation in CRs to be significantly associated with disease. These findings have spurred research on the biochemical, structural, and biological properties of CRs. 

In this review, we discuss recent progress on the role of *CHD8* in autism and neurodevelopment. To prepare this discussion, we firstly summarize key findings on the structural and biochemical features of *CHD8* as well as its relationship to other members of the *CHD* family. Following this, we reconsider present evidence for *CHD8* as a high confidence risk factor for ASD and as a genetic cause of a distinct neurodevelopmental syndrome highly associated with autism. Against this backdrop, we move on to examine the question of how various model organisms have helped to advance our insight into the biological role of *Chd8* in the developing and mature brain. We focus on the phenotypic effects of *Chd8* mutations and discuss transcriptional changes in relation to these phenotypes. Readers interested in the broader transcriptional function of *CHD8* are referred to previous reviews [7,8]. Finally, we consider continuing challenges for translational animal studies and how pluripotent stem cell-derived models could advance insight into the role of *CHD8* in autism. 

## 2. *CHD8* Is a Member of the CHD Family of CRs

The nucleosome is the basic subunit of chromatin and consists of a segment of DNA wound around eight histone proteins known as histone octamer. CRs regulate nucleosome sliding, conformational changes of nucleosomal DNA, and exchange of histone variants (reviewed in [9]). All of these processes influence chromatin configuration, and consequently, gene expression. Based on their biochemical activities, CRs are distinguished in those regulating post-translational modification of free histone tails and in those regulating histone-DNA contact within the nucleosome through ATP (adenosine triphosphate) hydrolysis.

**Figure 1:**
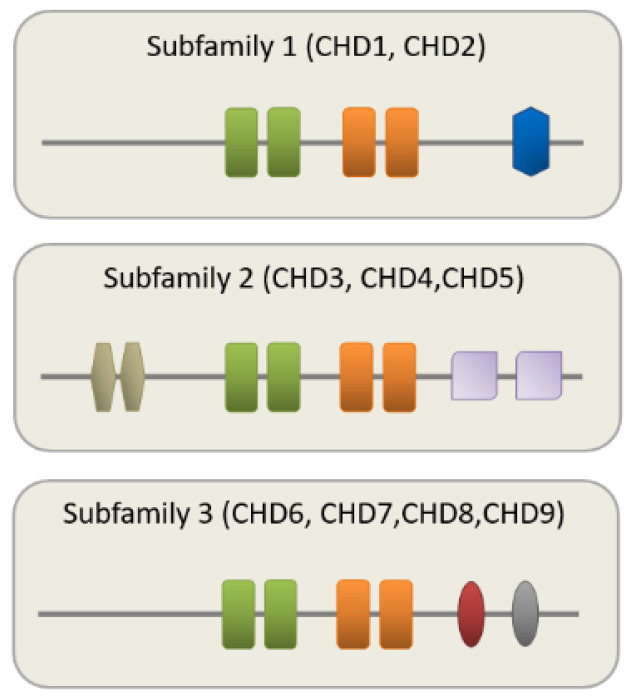
Schematic representation of the mammalian CHD family with conserved domains shown for each subfamily. The signature motif of the entire CHD family is an N-terminal tandem chromodomain (green boxes) responsible for chromatin binding. The central SNF2-family ATPase domain consists of two lobes (orange boxes) with each containing 2 tandem RecA-like folds parts known as DExx and HELIC. The ATPase domain uses ATP hydrolysis to guide toward translocation down the DNA minor groove. CHD1 and CHD2 contain a C-terminal DNA-binding domain (blue box) that is replaced by a pair of PHD zinc-finger-like domains (purple boxes) in CHD3 to CHD5. Additional PHD domains localize to the N-terminus (olive boxes). The third subfamily, consisting of CHD6 to CHD9, is in the C-terminus more variable and contains functional motifs like SANT (red oval) or BRK (grey oval) domains. SANT domains support association with histone tails, while the BRK domain is also found in several SWI/SNF complexes. Schematic adapted from [11], attribution CC BY.

The catalytic ATPase domain is conserved among CR families (including SWI/SNF (switch/sucrose-non-fermenting), ISWI (imitation switch), and INO80 (inositol requiring 80) and CHD), and uses ATP to promote translocation down the DNA minor groove (reviewed in [10]). Additional domains next to the ATPase domain are important to chromatin binding, interaction with specific histone modifications, and/or regulation of ATPase activity [9]. For example, CHDs contain an N-terminal tandem chromodomain mediating binding to methylated lysine residues in free histone tails (Figure 1). 

The CHD family is further divided into 3 subfamilies based on the presence or absence of additional domains. Subfamily 3, consisting of CHD6 to CHD9, contains variable functional motifs like the SANT (Swi3, Ada2, N-Cor, and TFIIIB) or BRK (Brahma and Kismet domain) domains in its C-terminus (Figure 1). Overall, this combinatorial use of regulatory domains within and across the CHD family suggests common and subtype specific roles in chromatin remodeling. Consistent with this hypothesis, the NuRD complex (a large protein complex that combines nucleosome remodeling with histone deacetylation) associates with different CHDs during neurodevelopment. Thereby, each CHD contributes to stage-specific functions ranging from proliferation to differentiation (reviewed in [12]).

## 3. Mutations in *CHD8* Associate with Autism and Macrocephaly

ASD comprises a group of neurodevelopmental disorders that share to varying degree clinical symptoms and genetic risk factors (reviewed in [13]). ASD affects about one in 100 people worldwide and is more prevalent in boys than in girls. Early dysfunction in communication and social interaction frequently concurs with restricted, repetitive behavior. Concurrently, ASD associates with ID (35%), language delay (50%), or epilepsy (5–15%) [14]. All of these impairments persist lifelong and greatly reduce the quality of life. 

Heritability for ASD in monozygotic twins is high (0.62–0.94), and siblings of affected individuals show a high risk for relative recurrence risk (10.1%) [13]. The genetic architecture of ASD is complex, with risk being conferred by many independent loci containing common and rare variants as well as by de novo mutations with genetic variation ranging from single nucleotide polymorphism (SNP) to chromosomal deletion/duplication or other rearrangements. All of these genetic factors can combine in small or large number in ASD. Thereby, their relative contribution is likely different in specific syndromes and subpopulations; e.g., a single sporadic presentation in an individual with no previous family history is likely to differ from multiple affected individuals in a family. 

*CHD8* is one of the most frequently mutated and most penetrant genes in ASD [7]. Large scale analysis of parent-child trios/quads through whole exome sequencing (WES) [15,16,17,18] and targeted resequencing [17,19,20,21,22,23,24] established heterozygote de novo mutations in *CHD8* as high confidence risk factor in ASD. These mutations included frameshift, nonsense, missense, translocation, single nucleic acid deletion, and splice site variant mutations and are predicted to lead to a loss of function.

Interestingly, patients with de novo mutation in *CHD8* frequently exhibited an unusual large head circumference [17,19]. This phenotype was also observed in patients carrying a balanced translocation disrupting *CHD8* [25]. Additional facial dysmorphisms consisted of prominent forehead and eyes, and posteriorly rotated ears. In support of these early observations, integrated analysis of 15 patients with disruptive *CHD8* mutation (13 de novo, one inherited, and one of unknown origin) revealed that autism was the most common diagnosis (*N* =13) closely followed by macrocephaly (*N* = 12) [20]. Orbital overgrowth developed already within the first 2 months postnatally, pointing to a neurodevelopmental origin, and associated with increased head growth throughout early childhood. Besides these core symptoms, patients with de novo *CHD8* mutation developed to a lesser degree recurrent obstipation and sleep disturbances. 

These landmark studies are supported by recent findings that strengthen the hypothesis that de novo mutations in *CHD8* underpin a distinct subtype in ASD. Another patient with a de novo balancing translocation disrupting *CHD8* presented with autistic features, developmental delay, and language disability at 2 years age [26]. Collated clinical data from individuals with deleterious *CHD8* mutations (*N* = 51) corroborated further the existence of a distinct ASD subtype in which children manifest developmental delay, ID, and/or ASD in addition to characteristic facies (see Figure 3 of [26]). 

Recent studies also sought to reassess the overgrowth phenotype in individuals with de novo *CHD8* mutations. Ostrowski et al. [27] identified 27 unrelated patients with pathogenic or likely pathogenic *CHD8* mutation (25 null variants, 2 missense variants). All of them showed ID with 85% in the mild or moderate range, and 85% had a height and/or head circumference ≥ 2 standard deviations above the mean. Behavioral symptoms were common (78%) with over half (56%) either diagnosed with ASD or autistic traits (see Table 1 of [27]). Similarly, An et al. [28] reported the identification of 4 new de novo *CHD8* mutations (including 2 nonsense variants, 1 splice variant, and 1 missense variant) in Chinese individuals with ASD. Comprehensive phenotyping revealed that these individuals shared prenatal onset macrocephaly, facial dysmorphism, overgrowth during puberty, ID, and sleep disorders (see Table 1 of [28]).

In a recent phenotype-to-genotype approach, Wu et al. [29] carried out WES on 67 families with ASD and abnormal head circumference. Pathogenic or likely pathogenic mutations were identified in 15% of the affected individuals with *CHD8* and *PTEN* (phosphatase and tensin homolog) top ranked among disrupted candidate genes. 

Moving beyond genetics, Beighley et al. [30] focused on biological pathways shared by multiple ASD-associated risk genes. Thereby, the researchers investigated clinical and behavioral features of individuals with ASD (see Table 1 of [30]) carrying disruptive mutations (i) in *CHD8* (*N* = 15), (ii) in genes regulated by *CHD8* (*N* = 22) or (iii) in genes unrelated to *CHD8* (*N* = 106). Subgroup (i) and (ii) shared less severe adaptive deficits in communication skills, similar functional language, more social motivation challenges in those with ASD, macrocephaly, higher weight, and lower seizure prevalence when compared to subgroup (iii). This suggests that neurodevelopmental subtypes defined by gene–gene interactions can improve stratification of patients relative to mutations alone.

In summary, compelling genetic and clinical evidence supports a role of de novo mutations in *CHD8* as genetic cause of a distinct subtype in ASD that includes the core symptoms of autism, macrocephaly, and facial dysmorphism. 

## 4. Chd8 Function in Neurodevelopment and Neuronal Function 

Different animal models have been used to investigate the function of *Chd8* during neurodevelopment and for mature brain function. In the context of this review, we will scrutinize how these experiments have helped to understand core symptoms of patients with *CHD8* mutations. We discuss these studies by chronological order and/or by common topics. To facilitate navigation through this section, key findings are summarized in a tabular format (Table 1). 

### 4.1. A Role of Chd8 in Embryonic Development 

Wingless (Wnt) signaling plays an important role for neurodevelopment, cellular proliferation, differentiation, and morphogenesis (reviewed in [31]). In the presence of Wnt ligands, cytoplasmatic β-catenin is stabilized and translocates to the nucleus, where it stimulates gene expression through binding to the DNA-bound TF TCF (T-cell factor). By means of a yeast two hybrid screen, Sakamoto et al. [32] identified *Chd8* as β-catenin binding partner. Dorsal injection of *Chd8* mRNA in *Xenopus* embryos inhibited axis formation, Wnt/β-catenin-dependent axis duplication, and ultimately, head formation. Contrariwise, antisense RNA-mediated blocking of *Chd8* splicing (>80%) in *Zebrafish* embryos led to an increase in interorbital distance, a surrogate measurement for head circumference [20]. This process associated with increased expression of chordin (necessary for forebrain development), of otx2 (early marker of midbrain/forebrain neural progenitor cells, NPCs), and of HuC/D (maker for newborn neurons). 

Together, these two studies suggest a role of *Chd8* in early head development and the proliferation of mid- and forebrain progenitors (Table 1). It is important to note, however, that both studies did not recapitulate heterozygote de novo mutations of *CHD8* in human. On the one hand, Sakamoto et al. [32] used overexpression of *Chd8* as opposed to loss of *CHD8* function in humans, while on the other hand, Bernier et al. [20] blocked regular splicing of *Chd8* by 80%. This effect-size is very likely to surpass the one expected from heterologous splice-site mutations of *CHD8* in human.

**Table 1:** Transgenic animal studies on *Chd8* function in the developing and mature brain.

Ref.	Model	System	Timepoint(s)	Tissue Type	Major Findings for Altered Chd8 Expression
[32]	*Xenopus*	*Chd8* mRNA injection	Stage 40–41	Whole embryo	Impaired β-catenin dependent axis duplication and head formation.
[20]	*Zebrafish*	*Chd8* splice-block- ing anti-sense RNA	Day 4.3 post- fertilization	Embryo head	Enhanced proliferation of midbrain and forebrain progenitor cells. Increased interorbital distance.
[33,34]	Mice (C57BL/6J)	LoxP-neo cassette replacing all exons	E: 5.5, 6.5, 7.5 8.5, 9.5		Embryonic growth retardation and lethality due to massive cell death.
[35]	Mice (Swiss Webster)	E13 shRNA electroporation	E: 15	GFP^+^ cortical cells	Impaired NPC proliferation and differentiation associates with **reduced dendritic arborization** and deficits in social behavior.
[36]	Mice (C57BL/6J)	Cre-LoxP Recombination Exon 11-13	E: 10, 12, 14, 16, 18; P: 91	Whole brain	Increased brain weight and ASD-like behavior. ASD risk genes and genes involved in synapse and ion channel function are downregulated. Up-regulation of the master regulator REST might underlie neuro- developmental delay.
[37]	Mice (C57BL/6J)	CRISPR/cas9 Exon 1 (7bp)	P: 70–84	Ctx, Stria, NAc, VTA, Hipp, Amyg, Hyp	Macrocephaly. Expression changes in genes related to ASD, neuro- development, cell adhesion, histone and chromatin modification. **Reduced local inhibitory signaling in the NAc leads to enhanced excitatory input on MSNs.**
[38]	Mice (C57BL/6J)	CRISPR/cas9 Exon 5 (5bp) Both sexes	E: 12.5, 14.5, 17.5; P: 0, 58	Bulk forebrain	Increased neocortical brain volume due to increased NPC proliferation. Cognitive deficits associate with expression changes in genes related to RNA processing chromatin remodeling, and cell cycle regulation.
[39]	Mice (C57BL/6J)	*Chd8^+^*^/−^, Exon 3 (*Chd8*^flox/+^ X *β-actinCre^+/−^*)	E: 12.5; P: 5, 26–27; W: 9–12, 15–18	Ctx, Hipp, Cer	Increased cortical, hippocampal, and cerebellar areas. Hypertelorism. Cell adhesion and axon guidance genes are downregulated postnatally. Increased long-range connectivity in entorhinal, retrosplenial, auditory cortical, and posterior hippocampal areas. Hyperactive pups show delayed motor development, while hypoactive adults show heightened interest in social cues.
[40]	Mice	Knock-in	E: 15.5, 18.5;	Ctx, Hipp	Male-preponderant abnormalities in social communication in pups, anxiety-like behavior in juveniles, and isolation-induced grooming in adults. **Opposite changes in excitatory and inhibitory neuronal firing in male and female hippocampi** associate with gene expression changes enriched in matrix- and synapse-related gene sets. ASD- and glia gene sets are enriched in male hippocampi only.
	(C57BL/6J)	*Chd8* ^+/N2373K^	P: 0, 7, 14		
				
[41]	Mice (mixed C57BL6;129Sc)	*Chd8*^+/−^ and *Chd8*^−/−^ Exon 4 (*Chd8*^flox/+^ X *Olig1-Cre^+/−^*)	E: 14.5 P: 1, 14	CC, ON, VWM, SC	Chd8 chromatin binding prevail in early OLs and promotes expression of lineage specific genes through recruitment of the methytransferase KMT2. Null-mice die with severe myelination defects, whereas hetero- zygote mice show circumscribed myelination deficits.
[42]	*Drosophila*	Hypomorph *kis^k13460^* and heteroallelic *kis^LM27^/kis^k13416^*	Third instar larvae	NMJ, CNS	Under sustained stimulation. Kis promotes presynaptic endocytosis at GSEAthe NMJ. Kis-dependent transcription supports restoration of the recycling vesicle pool.
[43]	Mice (C57BL/6J)	*Chd8^flox/+^* X *β-actin-* *Cre^+/−^* or *Nkx.1-Cre^+/−^* or *NEX-Cre^+/−^*	P: 5, 13–15, 19- 22, 55–60	Ctx (ex- vivo slices)	**Impaired synaptic development of prefrontal pyramidal neurons** in a stage-specific and cell-autonomous manner associates with **contrasting** **changes in excitatory and inhibitory synaptic transmission**. **Homeostatic plasticity** **is perturbed in heterozygote neurons.**
[44]	*C. elegans* (Bristol N2)	*CHD8•chd-7(gk306)*	96 h posthatch	NA	**Reduced synaptic vesicle recycling** associates with reduced habituation of response probability indicating reduced plasticity of neuronal circuits.

Abbreviations: Amyg, amygdala; CC, corpus callous; Cer, cerebellum; CNS, central nervous system; Ctx, cortex; E, embryonic day; GEP, gene expression profiling; GSEA, gene set enrichment analysis; Hipp, hippocampus; Hyp, hypothalamus; NA, not applicable, NAc, nucleus accumbens; NMJ, neuromuscular junction; ON, optic nerve; P, postnatal day; SC, spinal cord; Stria, striatum; VTA, ventral tegmental area; VWM, ventral white matter; w, week. Findings related to synaptic dysfunction are highlighted in bold.

Similar concerns on gene dosage refer to the results from mice carrying homozygous deletions of *Chd8* [33]. *Chd8* was well-expressed from early- to mid-stage mouse embryogenesis, however, null mice showed no signs of upregulation in Wnt target genes nor of embryonic overgrowth. On the contrary, null embryos exhibited from embryonic day 5.5 onward growth retardation and growth arrest. This phenotype concurred with wide-spread p53-mediated apoptosis [34]. A plausible explanation for this fatal outcome was the finding that *Chd8* formed with linker histone H1 and p53 a chromatin bound complex that prevented p53 transactivation in wild-type mice.

### 4.2. A Role of Chd8 for Neural Progenitors, Transcription, Neurotransmission, and RNA Splicing

Embryonic lethality of *Chd8* in null mice encouraged Durak et al. [35] to conduct in utero knock-down of *Chd8* to investigate its role during early neurodevelopment. *Chd8* downregulation reduced the proliferation of neural progenitor cells (NPCs) and led to the production of fewer neurons. Gene expression profiling (GEP) and chromatin immunoprecipitation sequencing (ChIP-seq) suggested that *Chd8* binding to the promoters of cell cycle genes enhanced their expression in NPCs. This result is in agreement with a number of independent studies on the role of *CHD8* and other CHD proteins in cell cycle regulation [45,46,47,48]. Interestingly, *Chd8*-deficient NPCs showed additionally deregulation of numerous known ASD risk genes, and of genes controlled by Wnt signaling and the Polycomb group (PcG) repressive complex (the role of PcG for neurodevelopment is reviewed in [49,50]). These findings led the authors to propose that *Chd8* promoted NPC self-renewal by transactivation of cell cycle genes and PcG-mediated downregulation of neural genes. 

Unexpectedly though, *Chd8* knock-down in cortical NPCs led to the downregulation of primary transducers and effectors of the Wnt signaling pathway (e.g., *Fzd1*, *Fzd2*, *Dvl2*, *Dvl3*, and *Ctnnb1*). Several of these targets were bound by *Chd8*, suggesting that *Chd8* was needed to sustain Wnt signaling during neocorticogenesis. Moreover, *Chd8* knock-down led to mislocalization of adult cortical neurons and reduced dendritic arborization [35]. Interestingly, behavioral tests revealed abnormal social interaction in adult knock-down mice that resembled some of the behavioral symptoms among individuals with *CHD8* mutation. In support of these findings, expression of a stabilized form of β-catenin rescued cortical development and behavioral abnormalities. 

Taken together, these results suggest that *Chd8* regulates in a stage-specific manner the proliferation and differentiation of NPCs, leading to impairments in corticogenesis and social behavior in *Chd8* knock-down mice (Table 1). Although this study firstly connected *Chd8*-dependent molecular and cellular processes with behavioral functions known to be disrupted in human disease, several caveats need to be considered: The researchers [35] assessed the efficiency of shRNA-mediated *Chd8* knock-down in 3 cell lines and reported a reduction of *Chd8* mRNAs by 50%. By contrast, the efficiency of *Chd8* knock-down in embryonic brain remained untested. Knock-down efficiency is very likely to decline over time due to shRNA degradation and cell division, leading to different levels of *Chd8* expression in early and late NPCs. Hence, it remains to be clarified to what degree cellular and behavioral anomalies in knock-down mice actually recapitulated pathogenesis in human disease. 

To track more closely *CHD8* mutations in patients, Katayama et al. [36] chose to use heterozygote *Chd8* knock-out mice lacking either both isoforms or only the long isoform of *Chd8*. Knock-out mice were viable and showed increased embryonic and adult brain weight, as well as increased adult brain volume when compared to wild-type littermates.

Behavioral tests revealed ASD-like symptoms including increased anxiety, repetitive behavior, and altered social behavior. At the molecular level, ChIP-seq analysis showed that *Chd8* was particularly enriched at ASD-related genes, the expression of which was downregulated in knock-out mice. Moreover, GEP of whole brains revealed small, global changes in gene expression reminiscent of those from individuals with ASD. Gene set enrichment analysis (GSEA) tests whether a list of genes ranked by fold change (usually covering the entire transcriptome) is enriched for pre-curated gene sets. This allows us to identify differences due to small, but coordinated, changes in a large number of genes. GSEA of *Chd8* knock-out brains revealed downregulation of genes known to be downregulated in patients with ASD, and of genes related to synapse or to ion channel function. 

Furthermore, GSEA suggested that neural development was delayed during the early to mid-fetal stage in *Chd8* knock-out mice, and that this delay may contribute to the ASD-like phenotype in adults. Expression of the enriched gene sets was unrelated to Wnt/β-catenin and p53; instead, it related to REST (RE-1 silencing factor), a master regulator of neuronal development that suppresses the transcription of neural genes. REST target genes were markedly enriched in mid-fetal stages, supporting the notion that aberrant REST activation in *Chd8* knock-out mice could delay their timely expression. Of note, postmortem brains from patients with ASD showed similar significant downregulations of REST target genes.

Taken together, heterozygote *Chd8* knock-out mice showed delayed neurodevelopment, brain overgrowth, and ASD-like behavioral symptoms (Table 1). Notably, despite the similarities in behavior, the larger brain size measured by Katayama et al. [36] appears in contradiction to the impaired corticogenesis reported by Durak et al. [35]. Besides likely differences in gene dosage, acute knock-down of *Chd8* may leave little space for compensation during the neurogenic period. Contrariwise, a *Chd8* germline mutation may enable a compensatory response that overcomes early developmental delay. Moreover, depletion of *Chd8* in transfected cells only may differ from a *Chd8* germline mutation, affecting uniformly all cells during neurodevelopment. 

This hypothesis is supported by another *Chd8* knock-out model for which Platt et al. [37] chose to use CRISPR/Cas9 editing to create a heterozygote frameshift mutation. In 10-week-old heterozygote mice, magnetic resonance imaging (MRI) showed an increase in total brain volume and in intraocular distance resembling findings in patients. *Chd8* was expressed in different cell types including (inter-) neurons, oligodendrocytes (see further below), and astrocytes. Owing to unaffected proliferation and migration of cortical NPCs, lamination and specification of these cells was unaffected in the somatosensory cortex of heterozygote mice. Behaviorally, heterozygote mice showed mild deficits in social interaction, elevated anxiety, and increased acquired motor learning. 

**Figure 2:**
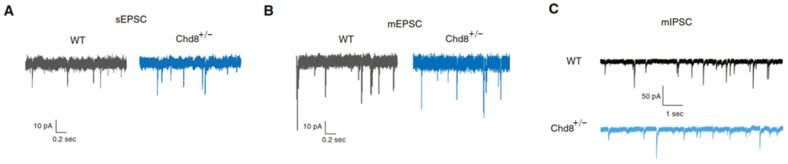
Schematic representation of synaptic dysfunction within MSNs in the NAc of *Chd8* heterozygote mice. (**A**) Representative sEPSC (spontaneous excitatory postsynaptic current) traces show an increase in sEPSC frequency and amplitude in *Chd8*^+/−^ mice relative to wild-type (*WT*) littermates. (**B**) Representative mEPSC (miniature excitatory postsynaptic current) traces show no difference in either mEPSC frequency or amplitude between *Chd8*^+/−^ and WT mice. (**C**) Representative mIPSC (miniature inhibitory current) traces show no difference between mIPSC frequency between genotypes albeit a decrease in mIPSC amplitude in *Chd8*^+/−^ mice relative to WT littermates. Schematic adapted from [37], attribution license 4985961341269.

GEP across multiple brain regions evidenced global deregulation of genes related to ASD, neurodevelopment, histone and chromatin modifications, and neuronal and synaptic adhesion. Interestingly though, *Chd8* inhibited Wnt signaling exclusively in the nucleus accumbens (NAc). Together with deregulated adhesion, inhibition of Wnt signaling led to a local decrease of inhibitory transmission that may contribute to the enhanced excitatory input onto MSNs (medium spiny neurons) in the NAc in *Chd8* heterozygote mice (Figure 2). 

An important insight from this study is that the role of *Chd8* differs by brain region and cell type in the developing and adult brain. As a case in point, reduced *Chd8* function specifically impaired striatal neurotransmission, indicating that the NAc is an important node for social behavior and ASD pathology (Table 1). 

### 4.3. A Complex Role of Sex for Chd8 Mutations

A long-standing puzzle in ASD is that disease develops 4 times more common in males than in females [51], including patients with *CHD8* mutations [24]. Yet, little is known about the mechanisms leading to male–female differences in ASD despite the fact that such knowledge could provide important clues to etiology [52]. To address this issue, several studies [38,39,40] have sought to investigate the role of heterozygote *Chd8* mutations in both sexes with a focus on macrocephaly, behavior, cell types and brain regions, and gene networks. 

Gompers et al. [38] applied CRISPR/Cas9 editing to generate mice containing a heterozygote frameshift deletion in exon 5 of *Chd8*. Morphometric and MRI analysis revealed increases in absolute and relative volume across cortical regions, hippocampus, and amygdala in heterozygote mice. These alterations associated with impairments in cognition only, while social interactions, white matter organization, and long-range connectivity were unaffected. 

GEP of forebrains from 4 developmental stages and adult mice showed widespread, subtle changes in gene expression in pathways related to chromatin remodeling, cell cycle, and neurodevelopment (partly due to perturbed RNA splicing) consistent with previous studies [36,37]. Additional network analysis evidenced changes in pathways disrupted in neurodevelopmental disorders and neuroimmune signaling. Moreover, a co-expression module with peak expression in early brain development comprising dysregulation of RNA processing, chromatin remodeling, and cell-cycle genes was enriched for *Chd8* promoter binding. Consistent with these predictions, proliferation of NPCs in the germinal cortical ventricular and subventricular zone was increased in *Chd8* heterozygote mice and led to significant alterations in cortical projection neuron production (more Pax6^+^ radial glia cells versus less Tbr2^+^ intermediate progenitors) that may contribute to macrocephaly. 

In summation, Gompers et al. [38] corroborate by and large the results on macrocephaly and gene expression from previous heterozygote knock-out studies [36,37]. Likewise, these three studies agree on the absence of repetitive behavior previously reported upon in utero knock-down of *Chd8* [35]. 

On a second look, however, heterozygote mice diverged substantially in the affected behavioral domains. Gompers et al. noted impairments in the cognitive domain alone as opposed to Katayama et al. [36], who noted additional impairments in the social domain. One possible explanation is the different genetic background of the knock-out lines (Gompers; C57BL/6J versus Katayama; C57NL/6N) that are known to exhibit distinct behavioral phenotypes. Other more general reasons include differences in the time interval (Gompers; 6–16 weeks vs. Katayama; 12–50 weeks) during which mice underwent testing, and how this was exactly done. On a cautionary note, Gompers et al. [38] studied both sexes, albeit without analyzing each sex separately, an important issue in light of potential sex differences (see below [40]), while Katayama et al. [36] assessed male mice only.

*Chd8* knock-out mice [36,37,38] have provided valuable information on cellular and molecular phenotypes. Yet, the mechanisms through which these alterations connect to behavior remained poorly understood. Behavior reflects higher level systems function including neurotransmission, neuronal and synaptic activity, and neuronal connectivity (for a comprehensive representation see [53]). Altered brain connectivity, characterized by local over-connectivity and long-range under-connectivity, is thought to underpin some of the behavioral phenotypes in ASD [54]. However, resting-state functional MRI (rsfMRI) studies in patients with ASD have provided conflicting results with evidence for both reduced and increased long-range synchronization in spontaneous brain activity (reviewed in [55]). Since this uncertainty possibly reflects genotypic heterogeneity in ASD, knock-out studies on ASD risk genes provide unprecedented opportunities to address this topic. 

For this purpose, Suetterlin et al. [39] generated *Chd8^flox/+^* mice, which were then crossed with an ubiquitously expressing *β-actin-Cre* line to delete the *loxP*-flanked exon 3 of *Chd8.* This caused an early frameshift and termination of translation thought to generate a protein that lacks all functional domains. Consistent with this prediction, levels of *Chd8* mRNA and protein expression were reduced by ~50% in telencephalic vesicles (embryonic day 12.5) and newborn mice brains (postnatal day 5).

In agreement with aforementioned studies, heterozygote *Chd8* male mice presented increased brain size (cortex, hippocampus, and cerebellum) together with hypertelorism. Interestingly, comprehensive behavioral testing of heterozygote mice of both sexes detected various behavioral anomalies that developed in an age dependent manner: As pups, heterozygote mice were hyperactive and delayed in motor development. Conversely, in adulthood, they became hypoactive, but without socio-communicative deficit, and showed heightened interest in social cues. However, no significant differences between sexes were found except forelimb grip strength [39]. This test is broadly used to asses motor function, though it may also embrace motivational factors. 

Midgestational gene expression was only mildly affected in male neocortices. By contrast, some 600 genes were differentially expressed in the early postnatal cortex, including genes with a role in cell adhesion and axon guidance that were among those most significantly downregulated. 

These changes in postnatal gene expression encouraged Suetterlin et al. to investigate long-range connectivity in mature brain networks with the help of rsfMRI. Thereby, synchronous fluctuations in blood-oxygen-level dependent (BOLD) signals in different brain regions were taken as an indication of their functional connectivity. In fact, heterozygote male *Chd8* mice exhibited increased BOLD signals in the entorhinal, retrosplenial, auditory cortical, and posterior hippocampal areas that may contribute to alterations in sensory processing and behavior.

Concluding, Suetterlin et al. propose that *Chd8*-dependent changes in postnatal gene expression might foretell over-connectivity in sensory brain areas relevant to behavior (Table 1). A limitation of this interesting hypothesis is the purely descriptive analysis of gene expression changes, imaging, and behavior. Future loss and gain of function experiments are needed to support the hypothesized correlation by more mechanistic insight. Relatedly, male and female mice should be analyzed across all experiments to account for possible sex-differences (see below [40]). In fact, the absence of major sex-specific differences in behavior does not exclude the existence of sex-specific differences in the underpinning neuronal circuits, cellular structures, and molecular networks. 

Irrespective of these caveats, the delay in early motor development in heterozygote *Chd8* mice is intriguing. Delayed motor milestones in toddlers predate and predict the emergence and severity of language deficits in later life [56]. Likewise, early motor delay has been observed in patients with *CHD8* mutations [57]. Moreover, heightened interest in social cues in *Chd8* heterozygote mice could correspond to increases in functional connectivity of the auditory cortex, and thus recapitulate auditory processing deficits and over-responsivity to sensory stimuli in patients with ASD. 

In a contemporary study, Jung et al. [40] generated mice with a heterozygote *Chd8* mutation matching a mutation in patients with ASD (Asn2371LysfsX2 in humans and Asn2373LysfsX2 in mice). This frameshift led to a reduction of *Chd8* mRNA by ~25% and of *Chd8* protein by ~50%, most likely to degradation of the truncated protein, in both male and female brains.

MRI showed that total brain volume and axonal projections were unaffected in either sex though local brain volume of the anterior cingulate, anterior commissure, and cerebellum was selectively increased in *Chd8* heterozygote female mice. In behavioral testing, heterozygote *Chd8* mice exhibited male-preponderant abnormalities of social communication in pups, of anxiety-like mother-seeking/attachment behavior in juveniles, and of isolation-induced self-grooming in adults. To elucidate the cellular basis of this phenotype, Jung et al. analyzed the expression of c-fos, a proxy to neuronal activity, in the hippocampus of pups under resting and maternal-separation conditions. Interestingly, neuronal activity was suppressed in heterozygote female mice under resting conditions, and rose to neuronal activity of wild-type mice following maternal separation. In contrast, heterozygote males showed normal resting neuronal activity, but enhanced neuronal activity in response to maternal separation. Hence, both sexes showed a robust response to maternal separation, though their outcome differed pending on the pre-existing neuronal activity. Consistent with these findings, synaptic inhibitory transmission was increased in female hippocampus, but decreased in male hippocampus. Even though, expression levels of excitatory and inhibitory synaptic proteins were unchanged in male and female *Chd8* heterozygote mice when analyzed in whole brain and eight subregions of the brain. Thus, the actual mechanism underpinning alterations in hippocampal synapse function remained unresolved. 

GEP of whole brain and hippocampus in juvenile females showed that differentially expressed genes (DEGs) were strongly enriched in extracellular-matrix (ECM) related genes that are known to modulate neuronal and synapse development. In juvenile male hippocampi, both up- and downregulated DEGs were highly enriched for *Chd8* binding genes, whereas in females only downregulated DEGs were *Chd8*-bound. Furthermore, GSEA revealed that ECM-related and synapse-related genes were enriched in heterozygote female and male mice, respectively, and that these sexually dimorphic enrichments may underpin opposite synaptic transmission and neuronal firing activity. 

Interestingly, both sexes exhibit at postnatal day P0 transcriptomic patterns partly mimicking those from individuals with ASD. Thereby, neuronal gene sets were negatively enriched at P0 in males, whereas females showed a mix of positive and negative enrichments. This pattern was sustained in male mice at P25 and showed additionally a positive enrichment for glia-specific gene sets, a pattern previously observed in ASD [58]. Conversely, females exhibited largely an opposite pattern at P25. 

Taken together, Jung et al. provide compelling evidence for a sex-specific role of *Chd8* including male-preponderant behavioral abnormalities and a multitude of sexually dimorphic changes at neuronal, synaptic, and transcriptomic levels (Table 1). This male-preponderance of behavioral abnormalities in mice is consistent with the ~4-fold higher incidence of neuropsychiatric symptoms in male carriers of *CHD8* mutations. Likewise, the absence of behavioral phenotypes in heterozygote *Chd8* female mice corresponds with the lack of behavioral deficits in 3 females with a mutation in *CHD8* (among 4 carries and one unknown sex) in a control population [24]. In this context it is also important to note that reduced baseline neuronal activity in female mice did not affect other functional domains such as locomotion, or repetitive and anxiety-like behavior. 

Sex-preponderant behavioral deficits in *Chd8* heterozygote mice concurred with sexually dimorphic synaptic transmission and neuronal firing in the hippocampus, a brain region strongly connected to various ASD-related brain regions (reviewed in [59]). Interestingly, behavioral changes were stronger in males than in females, whereas transcriptomic changes were stronger in females than in males. This raises the possibility that transcriptomic changes in females contributed to lower resting neuronal excitation. Factors like sex chromosomes and hormones are obvious candidates to drive such a protective response. However, recent findings suggest that sex-independent mechanisms, such as downregulation of neuronal genes and upregulation of glial genes, operate in male patients with ASD [58]. Along this line, quantitative brain-wide mapping of inhibitory cell types in mice identified no less than 11 sex-dimorphic brain regions characterized by different numbers of interneuron classes [60] that could modulate the impact of *Chd8* on specific brain circuits. In any way, future studies in transgenic *Chd8* mice and in females with *CHD8* mutations are needed to elucidate the cellular and molecular basis of the sex-specific role of *CHD8*. 

### 4.4. A Role of Chd8 in Oligodendrocytes

Impairments in myelination are the source of cognitive, behavioral, and motor deficits characteristic of degenerative disorders (e.g., multiple sclerosis and leukodystrophies) and of patients with ASD. Affected individuals frequently exhibit central white matter abnormalities including deficits in myelin content and compaction [61,62]. Likewise, one-third of the individuals with mutations in *CHD8* present variable degrees of ventriculomegaly and delayed myelination [63]. This finding raises the question of a role of *CHD8* in myelination. 

During neurodevelopment, NPCs produce oligodendrocyte precursor cells (OPCs) that proliferate and differentiate into mature oligodendrocytes (OLs). Chromatin modification and remodeling play a critical role in this process as exemplified by the BRG1-containing SWI/SNF complex that is necessary for the transition from *Olig1*^+^ lineage progenitors (or NPCs) to OLs. BRG1, in co-operation with the lineage-specific TF Olig2, activates *CHD7*, a paralogue of *CHD8*, to control OPC differentiation and the timing of myelination (reviewed in [64]). 

Temporal analysis of OPC differentiation showed that *Chd8*, in contrast to Chd7, was stronger expressed in early OPCs than in differentiating or mature OLs [41]. Consistent with this pattern, in vitro ChIP-seq showed stronger *Chd8* chromatin binding in OPCs than in mature OL. Conversely, Chd7 chromatin binding prevailed in mature OLs and concurred with Brg1 co-occupancy. A large fraction of the *Chd8* binding sites was not shared by Chd7 at either stage (but see below [65]), indicating that *Chd8* targeted unique sites during OL development. Interestingly, *Chd8* bound to the promoters of genes encoding essential components of the BRG1-associated SWI/SNF complex (BAF) including *Brg1*, which is known to activate Chd7 expression during OPC differentiation [66]. Hence, *Chd8* could trigger a sequence of chromatin remodeling processes by linking Brg1 to Chd7 to promote OL lineage progression. 

To investigate the role of *Chd8* in vivo, Zhao et al. [41] decided to use mice with an OL-specific deletion of *Chd8* (floxed exon 4) in primitive OL progenitors (Pri-OPCs). Homozygous knock-out mice died at 3 weeks postnatally with severe myelination defects, whereas heterozygote mice were viable, though they presented significant myelination deficits in the optic nerve, spinal cord, corpus callosum, and to a lesser degree, in the cerebral cortex and cerebellum. In support of a cell-autonomous effect, tamoxifen-inducible postnatal deletion of *Chd8* in OPCs, but not in neurons, caused similar myelination defects. 

**Figure 3:**
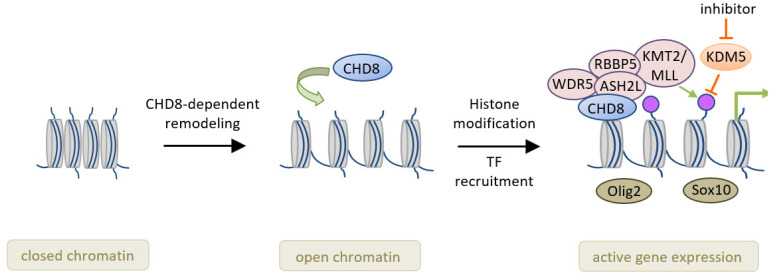
Schematic model for the role of *CHD8* in oligodendrocyte development. Closed chromatin in oligodendrocyte precursors cells prevents transcription (**left**). Upon binding, *CHD8* remodels chromatin into an open state (**middle**). This process provides access to lineage-specific transcription factors such as the oligodendrocyte factors Olig2 and Sox10. Following binding to their sequence-specific DNA-elements, these factors drive expression of target genes important to the progression of oligodendrocyte development. Concurrently, *CHD8* recruits the histone methyltransferase complex KMT2 that catalyzes histone 3 lysine 4 trimethylation (violet dots) that flags actively transcribed and poised gene promoters. Inhibition of the KDM5 subfamily of histone demethylases partially compensates differentiation defects in *Chd8* null mice (**right**). Schematic adapted from [41], attribution license 4838110791658.

Downregulated genes in purified *Chd8* null OPCs were enriched in those with a role in OL differentiation and cholesterol biosynthesis, whereas differentiation-inhibition genes, such as those encoding hedgehog- and p53-signaling, as well as Wnt/β-catenin signaling, were upregulated. Concurrently, histone methyltransferase KMT2/MLL target genes were downregulated in *Chd8* null OPCs. KMT2 catalyzes histone 3 lysine 4 trimethylation that flags pre-active gene promoters. In fact, *Chd8* recruited the KMT2 complex and thus activated the transcriptional program necessary for OPC differentiation. Consistent with this scenario, chromatin sites bound by *Chd8* were significantly enriched for consensus binding motifs for transcriptional regulators including Olig2 and Sox10 (Figure 3). Well-fitting, inhibition of the KDM5 subfamily of histone demethylases partially rescued differentiation defects in *Chd8* null OPCs in vitro and in vivo. 

Overall, the findings of Zhao et al. [41] strongly support a role of *Chd8* in chromatin remodeling during OL lineage-specific gene expression. 

However, *Chd8* deficiency did not affect the initial formation of OPCs, and myelination deficits were confined to specific regions of the developing brain. Such site-specificity could point to compensation from *CHD7*, which interacts and overlaps in expression with *CHD8* [67]. 

In support of this hypothesis, Marie et al. [65] provided experimental evidence for an interplay between *CHD8* and *CHD7* in the oligodendrocyte lineage. In OPCs, *Chd7* bound to genes involved in proliferation (such as *Cdk4* and *Cdk6*) and apoptosis (such as *p53*), while myelin-genes (such as *Mobp* and *Omg*) were bound only in OLs. Thereby, *Chd7* repressed proliferation/apoptosis genes and activated differentiation genes. Notably, *Chd7* and *Chd8* bound to the same regulatory regions of this subcategory of genes by acting together or compensating for each other. Consistent with this model, integration of genome-wide binding sites and associated epigenetic marks showed that *Chd7*/*Chd8* and lineage-specific TF like Olig2/Sox10 cooperate in the activation of oligodendroglia stage-specific genes. 

Taken together, these two studies [41,65] suggest that combinatorial interactions of *CHD8* with lineage-specific TFs (e.g., Olig2), chromatin modifiers (e.g., KMT2), and chromatin remodelers (e.g., *CHD7*) orchestrate the temporal and spatial control of OL lineage-specific gene regulation. Mutations in *CHD8* could disrupt this pathway and thus offer a plausible explanation for myelination defects in patients. 

### 4.5. A Role of Chd8 in Synaptic Vesicle Recycling 

The question of how *CHD8* may influence the activity of mature neurons has so far received little attention when compared to its role in neurodevelopment. Therefore, Latcheva et al. [42] sought to investigate the role of the *Drosophila* protein Kismet (Kis), the homologue to the mammalian CHD III subfamily, for mature synapse function. 

Neuronal function critically depends on endocytosis, a process by which membrane and synaptic proteins are recycled via different routes to meet the high volume of neurotransmitter vesicle turnover necessary to elicit postsynaptic responses. Early endosomes rapidly recycle cargo proteins back to the plasma membrane where they are internalized. Alternatively, early endosomes are trafficked to become recycling endosomes, which are slower to return internalized cargo to multiple locations on the cell surface (reviewed in [68]). Partitioning of endosomes into these pathways is regulated at each step by different Rab-family GTPases. Disruption at any step of this pathway is likely to converge on similar phenotypes at the *Drosophila* neuromuscular junction (NMJ), including overgrowth and an excess of satellite synaptic connections, termed boutons. Owing to the highly stereotypical development and plasticity of these boutons, the *Drosophila* NMJ presents a robust readout to endocytic defects. In addition, as a glutamatergic system, the *Drosophila* NMJ is a suitable model of glutamatergic neurotransmission in vivo.

Because homozygous *kis* null mutants are embryonically lethal, Latcheva et al. [42] chose to use adult viable hypomorph (*kis^k13416^*) and heteroallelic (*kis^LM27^/kis^k13416^*, constructed with the null allele, *kis^LM27^*) fly lines. Analysis of embryonic larvae showed that Kis promotes endocytosis by altering the expression of genes controlling the recycling of endocytic vesicles. Mutations in *kis* led to a significant increase in satellite boutons at the NMJ and a decrease in glutamatergic neurotransmission. Synaptic vesicles are distinguished in a readily releasable pool that is docked at the active zones and undergo release in response to physiological stimulation, and a recycling pool of vesicles that is recruited to sustain prolonged neurotransmission. Levels of glutamatergic vesicles (VGLUT) were barely affected under resting conditions in *kis* mutants. By contrast, responses after high frequency stimulation, which requires replenishment of the releasable pool from the recycling pool, were significantly reduced. Consistent with this finding, Rab11, a marker of recycling vesicles, was significantly less expressed at the boutons in *kis* mutant flies. In whole nervous system tissue, *Kis* chromatin binding was enriched at the endocytic genes *dap160* and *endoB*, which were significantly lower expressed at the NMJ in *kis* mutant flies. 

Taken together, Latcheva et al. [42] showed that Kis supports presynaptic endocytosis at the NMJ. Reduced levels of Rab11 were a likely source of altered trafficking and targeting of endosome vesicles in *kis* mutants that could influence synaptic vesicle cycling as well. Still, future studies are needed to answer how endosomes contribute to synaptic vesicle formation and to corroborate these findings in model systems more pertinent to synapse function in the human brain.

### 4.6. A Role of Chd8 in Homeostatic Plasticity and Habituation

ASD is associated with mutations in many genes that can affect the ratio between neuronal excitation and inhibition (reviewed in [69]). Yet, the impact of these mutations on network activity is frequently counterbalanced by homeostatic mechanism of plasticity. These serve to keep levels of excitatory and inhibitory activity (E/I balance) within an optimal range through changes in synaptic strength and intrinsic excitability (reviewed in [70]). As a result of this compensation, it becomes difficult to separate initial deficits from homeostatic responses: Many changes in E/I balance associated with mutations in ASD-risk genes have been therefore subsumed under either compensatory or maladaptive homeostatic mechanisms [69,71]. 

To address this uncertainty, Ellingford et al. [43] analyzed prefrontal deep layer (V/VI) pyramidal neurons during postnatal development of *Chd8* heterozygote mice [39]. Whole-cell voltage clamp recording of ex vivo slices (P20) showed a decreased frequency and amplitude of miniature excitatory postsynaptic currents (mEPSCs) versus an increased frequency of miniature inhibitory postsynaptic currents (mIPSCs). By contrast, neuronal intrinsic excitability, size, ion channel composition, and dendritic arborization of heterozygote neurons were unaffected. Consistent with this finding, temporal analysis of synapse development, including neonatal (P5), adolescent (P14), and adult (P55–60) mice, showed that *Chd8* heterozygosity affected different aspects of mEPSCs and mIPSCs in a stage-specific manner. Concurrent investigation of dendritic spine and vesicular GABA (γ-aminobutyric acid) transporter (VGAT)-positive synapse densities indicated that the decrease in mEPSC frequency at P20 was not the result of reduced excitatory synaptic density, but of reduced glutamate release probability. Contrariwise, the increase in mIPSC frequency associated with an increase in the number of inhibitory synapses. These contrasting synaptic phenotypes developed through cell autonomous mechanisms as evidenced by the use of excitatory neuron (*NEX-cChd8^+/^*^−^) or inhibitory neuron specific (*Nkx2.1-cChd8^+/^*^−^) heterozygote *Chd8* knock-out mice. 

Having established the synaptic phenotype of *Chd8* heterozygote cortical neurons, Ellingford et al. went on to investigate homeostatic responses in acute ex vivo slices. These preparations were incubated with TTX (tetrodotoxin, a selective inhibitor of Na^+^ channel conductance that blocks the generation of action potentials) and APV (2-amino-5-phosphonovalerate, a selective NMDA receptor antagonist) to reduce network activity. This treatment elicited an increase in mEPSC frequency in P13–15 wild-type, but not in *Chd8* heterozygote, cortical neurons. Contrariwise, incubation with TTX and Gabazin (6-Imino-3-(4-methoxyphenyl)-1(6H)-pyridazinebutanoic acid hydrobromide, a competitive and selective GABAA antagonist) triggered an increase in mIPSC frequency in *Chd8* heterozygote, but not in wild-type, neurons. 

In sum, *Chd8* heterozygosity affected the synaptic development of deep layer cortical neurons in a stage-specific and cell-autonomous manner that resulted in contrasting changes in excitatory and inhibitory synaptic transmission. Furthermore, *Chd8* heterozygote neurons reacted with increased inhibitory synaptic transmission upon blockade of spontaneous neurotransmission. 

These results significantly corroborate and extend previous studies. Platt et al. [37] reported unchanged excitatory and reduced inhibitory transmission in the NAc of adult *Chd8* heterozygote mice. Relatedly, Jung et al. [40] detected unaltered synaptic transmission in the cortex, but opposite changes in excitatory and inhibitory neuronal firing in male and female hippocampi. Together, these studies [37,40,43] suggest a critical role to *Chd8* in synaptic transmission in a manner dependent on the specific brain region, developmental stage, and sex under investigation. 

This critical role of *Chd8* applied also to homeostatic plasticity. Rapid changes in mEPSC amplitude, termed homeostatic scaling, have been originally described in cultures of hippocampal neurons following blockade of spontaneous glutamate transmission through NMDA receptors (reviewed in [70]). In the present study, Ellingford et al. [43] identified a new form of homeostatic plasticity, where blockade of spontaneous GABA transmission induced increased inhibitory synaptic transmission in *Chd8* heterozygote, but not in wild-type, neurons. This result suggests that homeostatic mechanisms of plasticity themselves are dysregulated in a mouse model of *Chd8* heterozygosity. In agreement with this view, Tatavarty et al. [72] recently showed that homeostatic mechanisms of plasticity are impaired in neuronal cultures with reduced *Shank3* (a high confidence ASD-risk gene) expression and in the primary visual cortex in *Shank3* knock-out mice. Future studies are warranted to unravel the molecular mechanism underpinning dysregulation of homeostatic mechanisms of plasticity and to translate these findings into human model systems. 

Despite remarkable progress at the level of single genes, as discussed here for *CHD8*, a major challenge in the field of ASD is the large and increasing number of candidates of known or unknown function, and the question of how they converge on genetic networks underpinning disease. This said, we keep our focus on the role of *Chd8*, when discussing recent progress from a systematic analysis of ASD-associated risk genes in *Caenorhabditis elegans*. McDiarmid et al. [44] used a custom made machine vision system to quantify 26 phenotypes spanning morphology, baseline locomotion, tactile sensitivity, and habituation learning in 135 strains of *C. elegans.* Each strain carried a mutation in a predicted ortholog of an ASD-associated risk gene (the symbol “•” is used to represent the human-to-*C. elegans* ortholog relationship of interest, e.g., *CHD8•chd-7(gk306)*). This analysis enabled the identification of hundreds of shared and unique genotype relationships. Among these, we discuss those derived from an automated short-term habituation learning behavioral paradigm that served as a sensitive in vivo assay of synaptic function and behavioral plasticity. 

Hierarchical clustering of similar phenotypes in sensory and habituation learning features identified 2 high-confidence gene clusters characterized by reduced habituation of response probability and hyperresponsivity to mechanosensory stimuli. The genes captured within these clusters were further selected for epistasis analysis. Crossing strains within the same cluster revealed a functional interaction between *CHD8•chd-7(gk306)* and *GAPVD1•rme-6(b1014)*, a guanine nucleotide exchange factor (GEF) (reviewed in [73]) that regulates endocytosis via activation of Rab5 (see [42]). The impairment in habituation of response probability of double mutants was equal to single mutants, indicating that they operate in the same genetic pathway to mediate short-term behavioral plasticity. In support of this finding, crossing of additional strains of *C. elegans* that contained distinct loss-of-function alleles of *CHD8•chd-7(gk209)* and *GAPVD1•rme-6(tm6649)* exhibited the same phenotypic profile, thus strengthening the genotype-to-phenotype relationship between *CHD8* and *GAPVD1*.

Additional crossing experiments between the 2 gene clusters included *CHD8•chd-7(gk306)* and *NLGN1/2/3/4X•nlg-1(ok259)*, the sole *C. elegans* ortholog of neuroligins in vertebrates. Notably, these double mutants displayed additive impairments in habituation, indicating that these two genes operate in parallel genetic pathways. Neuroligins are central to the establishment, properties, and dynamics of synapses by shaping the input/output relations of their resident neural circuits (reviewed in [74]). Consistent with this function, mutations in neuroligins have been associated with ASD and schizophrenia. 

Furthermore, synthetic lethal interactions were discovered between *CHD8•chd-7(gk306)* and *CTNNB1•bar-1(ga80)*, the *C. elegans* ortholog of β-catenin in vertebrates. This result strengthened previous evidence that *CHD8* fulfills additional roles in (neuro-) development beyond inhibition of Wnt/β-catenin signaling [32]. 

Overall, McDiarmid et al. firstly identified for *CHD8* a role in sensory habituation. This process refers to the plastic ability of a neural circuit to decrease responding to repeated sensory stimuli. Loss of *CHD8* specifically impaired habituation of response probability pointing to impaired plasticity in the decision of a neural circuit to respond rather than to respond vigorously. Interestingly, abnormalities in tactile sensitivity occur in >95% of individuals with ASD and their degree of habituation impairment correlates with the severity of social impairment (reviewed in [75]).

Moreover, this study highlights interactions among ASD-associated genes that would be missed by focusing solely on current high confidence risk genes (e.g., *CHD8* or *NLGN1*). The relevance of the genetic relationship between *CHD8*, a high confidence risk gene, and *GAPVD1*, a low confidence risk gene, is supported by recent observations implicating *CHD8* itself into endocytic pathways [42]. 

## 5. Discussion and Outlook

Translational animal models have provided a wealth of information on the critical role of *Chd8* in the developing and mature central nervous system (Table 1). There remain, however, notable differences between the outcomes of these studies and the degree to which they match human disease. An obvious issue at stake are differences owing to the experimental design. Early studies using overexpression, knock-down, or homozygous deletion of *Chd8* are most likely confounded by issues of gene-dosage and cellular mosaicism. Although later heterozygote *Chd8* knock-out mice have enabled to address these concerns, consistent measures of *Chd8* transcript levels with RNA-seq and protein levels with standardized antibodies and methodology across various developmental stages and tissues remain desirable to facilitate comparison between current findings. Such measures can also help distinguishing between phenotypic effects due to differences in gene dosage versus those due to differences in genetic background. 

In addition, transcriptomics needs to progress from the analysis of bulk tissues to single-cell sequencing techniques. *Chd8* is broadly expressed in various brain regions and cell types; however, not all of these may be affected in case of mutations (Table 1). Analysis of bulk tissues bears the risk to dismiss more confined changes during sensitive time windows of development. While such changes may be only transient, repercussions on brain development may be lasting owing to the dynamic expansion of early cell populations. Thus, the identification of early cell populations particularly vulnerable to *Chd8* heterozygosity can provide a basis to systemic dysfunction of the mature brain. For instance, cell-lineage-specific ablation of *Chd8* has uncovered important cell-autonomous roles of *Chd8* during oligodendrocyte development [41] and neurotransmission [43]. While this kind of experiments have provided important insights for the role of *Chd8* in cell specification and differentiation, it is important to stay aware that healthy brain function reflects the intricate balance of interactions between neurons, astrocytes, microglia, and vascular cells, which become compromised during disease. Therefore, global and tissue-/cell-type specific heterozygote *Chd8* knock-out models are equally necessary and do complement each other. 

In general, transgenic mice studies need to explore sex-specific differences more explicitly. This requires the establishment of a catalogue of standardized operating procedures for behavioral testing to make results from different laboratories more comparable. 

Synaptic dysfunction is thought to be one of the key pathophysiological mechanisms underpinning behavioral symptoms in ASD and other neurodevelopmental disorders. Interestingly, heterozygote *Chd8* mice provide preliminary evidence (Table 1, highlighted in bold) to support this hypothesis: Platt et al. [37] reported a local decrease of inhibitory transmission that may contribute to the enhanced excitatory input onto MSNs in the NAc. On the other hand, Jung et al. [40] found that synaptic inhibitory transmission was increased in female hippocampus, but decreased in male hippocampus; however, expression levels of excitatory and inhibitory synaptic proteins were unchanged in either sex. Interestingly, Ellingford et al. [43] described changes in excitatory and inhibitory synaptic transmission in pyramidal cortical neurons that associated with disrupted mechanisms of homeostatic plasticity. While it is tempting to speculate that these findings might apply to individuals with mutations in *CHD8*, we are not aware of any study on human brain tissues to support this hypothesis. In this respect, pluripotent stem cell-derived cerebral organoids (see below [76]) could help to bridge the gap between mice and human studies. 

At the system scale, model systems like *Drosophila* or *C. elegans* have been used to uncover the effect of *CHD8* disruption on different brain functions including neurotransmission [42] and neuronal plasticity [75]. Along this line, Jin et al. [77] most recently reported a scalable genetic screen of a panel of high confidence ASD risk genes, including *CHD8*, in the developing mouse cortex. Following infection in utero of lentiviral CRISPR/Cas9 guide RNA libraries, the effects of the introduced frameshift mutations in these risk genes was analyzed by single cell RNA-sequencing of perturbed cells in the postnatal brain. These experiment revealed that perturbations in *Chd8* might alter oligodendrocyte maturation in agreement with previous finding [65]. While this kind of genetic screen might cast new light on genetic networks underpinning high confidence ASD risk genes [78], one obvious limitation of this approach is that it does not extend to phenotypic analysis, nor to high level system dysfunction in ASD.

Besides the aforementioned issues, future studies need to address a number of outstanding questions. Firstly, heterozygote *Chd8* mutations in mice seemed to be associated with rather mild behavioral abnormalities when compared to patients. One plausible explanation is that most transgenic studies do not directly recapitulate human *CHD8* mutations that differ in location and, most likely, in biological activity. *CHD8* mutations are predicted to encode different kind of loss-of-function (haploinsufficiency, hypomorphic, or dominant effects on protein function) and may associate with distinct symptoms. For example, patients carrying specific mutations leading to premature stop codons (i.e., S62X and E1114X) share autistic core symptoms. Contrariwise, the patient with the mutation S62X does not develop macrocephaly and ID [20,30], suggesting that the S62X mutation manifests only a subset of the clinical phenotypes. Designing *Chd8* mutations in mice therefore needs to consider and match as closest as possible the human condition and sharpen the tools for mice analysis in parallel. As pointed out before, recent clinical studies [26,27,30] have sought to better define the relations or penetrance of phenotypes among carries of *CHD8* mutations. To harvest the full potential of transgenic animal studies, it will be mandatory to intensify interactions between clinical and basic research. 

Secondly, ‘humanized’ mice models will not only enhance the clinical relevance of the observed phenotypes (Table 1), but could also provide valuable models for tailored therapies [79]. In this regard, current studies have sought to ameliorate abnormal phenotypes in *Chd8* knockdown or knockout mice mainly through overexpression experiments. For example, Durak et al. [35] have carried out in utero electroporation of a stabilized form of β-catenin to rescue the effects of *Chd8* knockdown during neocorticogenesis. While this approach is inapplicable in humans, it also raises concerns in light of the tissue-specific role of *Chd8* in the regulation of wingless signaling [37]. More promisingly, Zhao et al. [41] showed that pharmacological inhibition of the KDM5 subfamily of histone demethylases in vivo rescued in part oligodendrocyte differentiation defects in *Chd8* null mice (Figure 3). Even then, we would like to caution that undesired side effects of this treatment on brain function are likely owing to the eminent role of epigenetic processes in neurodevelopment and mental disease (reviewed in [80,81]). Overall, future studies need to pay more attention to both tailored interventions in ‘humanized’ mice models and to their clinical applicability. 

Thirdly, future studies need to consider principal differences in brain development between mice and men (reviewed in [82]). The neocortex is the home of higher cognitive functions and, in evolutionary terms, is the youngest part of the mammalian brain. Since its origin, the neocortex has expanded in several mammalian lineages, and this is particularly notable in humans. This expansion reflects an increase in the number of neocortical neurons, which is determined during development and primarily reflects the number of neurogenic divisions of distinct classes of neural progenitor cells. 

Human pluripotent stem cell-derived brain cells could help to overcome this species gap, at least in part. Embryonic stem cell (ESC) and induced pluripotent stem cell (iPSC) derived neuronal cell types exhibit genetically encoded molecular and cellular phenotypes matching human mid- to late-term brain development. Most recently, Villa et al. [76] reported the effect of *CHD8* mutations on human cortical development by combining the generation of ESC-derived human cerebral organoids with single cell transcriptomics. By adopting an isogenic design of patient-specific mutations, wild-type and mutated *CHD8* conditions were investigated on identical genetic backgrounds. Interestingly, *CHD8* haploinsufficiency caused an accelerated generation of inhibitory neurons and a delayed generation of excitatory neurons as evidenced by transcriptomics and cellular assays. This imbalance resulted in an extension of the proliferative phase and a significant enlargement of cerebral organoids. In an elegant control experiment, ESCs engineered to harbor the *CHD8* mutation S62X (see above) gave rise to cerebral organoids indistinguishable in size to those derived from wild-type ESCs, thus aligning with the absence of macrocephaly in human carriers. 

Ultimately, these interesting findings spur the question of whether pluripotent stem cell-derived models could be used additionally for the analysis of higher levels of brain functions. In support of this concept, ESC/iPSC-derived cells can be transplanted at different stages of development in mice brains where they integrate to a different degree. This allows capturing more advanced stages of differentiation, including the generation of most cell types of the brain and the formation of complex cell–cell interactions including vascularization and synaptic pruning. Then, cellular abnormalities, including altered neurotransmission and network activity, can be assessed in a more naturalistic environment that may be more permissive to disease-relevant phenotypes. For example, this approach has allowed us to assess iPSC-derived cells from healthy controls and patients with schizophrenia for various phenotypes ranging from myelination to behavior (reviewed in [83,84]). Highly penetrant mutations in *CHD8* might make an even better case for ESC/iPSC-based disease modeling than highly polygenic diseases like schizophrenia. Nevertheless, caution still needs to be applied to extrapolate from these reductionist models to potential alterations in the living brain of patients.

All in all, the need to understand and develop therapies for ASD persist. The identification of *CHD8* as a high confidence risk factor for ASD and genetic cause of a distinct syndrome has compelled studies from biochemistry to animal models to pluripotent stem cells. Today, scientists are equipped with unpresented tools. Beyond further refinements in the design and analysis of animal models, joining the forces and expertise from different and newly emerging fields will be key to progress on *CHD8* function in mental health and disease. 

## Abbreviations

ASD	autism spectrum disorder
ATP	adenosine triphosphate
CHD	Chromodomain Helicase DNA binding
ChIP-seq	chromatin immunoprecipitation sequencing
CR	chromatin remodeler
DEG	differentially expressed genes
ECM	extracellular matrix
ESC	embryonic stem cell
GEP	gene expression profiling
GSEA	gene set enrichment analysis
GWAS	genome-wide association studies
ID	intellectual disability
iPSC	induced pluripotent stem cell
Kis	Kismet
NMJ	neuromuscular junction
MRI	magnetic resonance imaging
NAc	nucleus accumbens
NPC	neural progenitor cell
OPC	oligodendrocyte precursor cell
OL	oligodendrocyte
p53	tumor suppressor gene p53
PcG	Polycomb group complex
TF	transcription factor
WES	whole exome sequencing
Wnt	wingless

## References

[1] Tyagi M., Imam N., Verma K., Patel A.K. (2016). Chromatin remodelers: We are the drivers!. Nucleus.

[2] Jaenisch R., Bird A. (2003). Epigenetic regulation of gene expression: How the genome integrates intrinsic and environmental signals. Nat. Genet..

[3] Zhang T.-Y., Meaney M.J. (2010). Epigenetics and the environmental regulation of the genome and its function. Annu. Rev. Psychol..

[4] Murgatroyd C., Wu Y., Bockmühl Y., Spengler D. (2010). Genes learn from stress: How infantile trauma programs us for depression. Epigenetics.

[5] Murgatroyd C., Spengler D. (2012). Genetic variation in the epigenetic machinery and mental health. Curr. Psychiatry Rep..

[6] Krumm N., O’Roak B.J., Shendure J., Eichler E.E. (2014). A de novo convergence of autism genetics and molecular neuroscience. Trends Neurosci..

[7] Barnard R.A., Pomaville M.B., O’Roak B.J. (2015). Mutations and Modeling of the Chromatin Remodeler CHD8 Define an Emerging Autism Etiology. Front. Neurosci..

[8] Wade A.A., Lim K., Catta-Preta R., Nord A.S. (2018). Common CHD8 Genomic Targets Contrast With Model-Specific Transcriptional Impacts of CHD8 Haploinsufficiency. Front. Mol. Neurosci..

[9] Clapier C.R., Cairns B.R. (2009). The biology of chromatin remodeling complexes. Annu. Rev. Biochem..

[10] Singleton M.R., Dillingham M.S., Wigley D.B. (2007). Structure and mechanism of helicases and nucleic acid translocases. Annu. Rev. Biochem..

[11] Hu Y., Lai Y., Zhu D. (2014). Transcription regulation by CHD proteins to control plant development. Front. Plant Sci..

[12] Hoffmann A., Spengler D. (2019). Chromatin Remodeling Complex NuRD in Neurodevelopment and Neurodevelopmental Disorders. Front. Genet..

[13] Geschwind D.H., State M.W. (2015). Gene hunting in autism spectrum disorder: On the path to precision medicine. Lancet Neurol..

[14] American Psychiatric Association (2013). Diagnostic and statistical manual of mental disorders: DSM-5. Development.

[15] Iossifov I., Ronemus M., Levy D., Wang Z., Hakker I., Rosenbaum J., Yamrom B., Lee Y.-H., Narzisi G., Leotta A. (2012). De novo gene disruptions in children on the autistic spectrum. Neuron.

[16] Neale B.M., Kou Y., Liu L., Ma’ayan A., Samocha K.E., Sabo A., Lin C.-F., Stevens C., Wang L.-S., Makarov V. (2012). Patterns and rates of exonic de novo mutations in autism spectrum disorders. Nature.

[17] O’Roak B.J., Vives L., Girirajan S., Karakoc E., Krumm N., Coe B.P., Levy R., Ko A., Lee C., Smith J.D. (2012). Sporadic autism exomes reveal a highly interconnected protein network of de novo mutations. Nature.

[18] Sanders S.J., Murtha M.T., Gupta A.R., Murdoch J.D., Raubeson M.J., Willsey A.J., Ercan-Sencicek A.G., DiLullo N.M., Parikshak N.N., Stein J.L. (2012). De novo mutations revealed by whole-exome sequencing are strongly associated with autism. Nature.

[19] O’Roak B.J., Stessman H.A., Boyle E.A., Witherspoon K.T., Martin B., Lee C., Vives L., Baker C., Hiatt J.B., Nickerson D.A. (2014). Recurrent de novo mutations implicate novel genes underlying simplex autism risk. Nat. Commun..

[20] Bernier R., Golzio C., Xiong B., Stessman H.A., Coe B.P., Penn O., Witherspoon K., Gerdts J., Baker C., Vulto-van Silfhout A.T. (2014). Disruptive CHD8 mutations define a subtype of autism early in development. Cell.

[21] De Rubeis S., He X., Goldberg A.P., Poultney C.S., Samocha K., Cicek A.E., Kou Y., Liu L., Fromer M., Walker S. (2014). Synaptic, transcriptional and chromatin genes disrupted in autism. Nature.

[22] Iossifov I., O’Roak B.J., Sanders S.J., Ronemus M., Krumm N., Levy D., Stessman H.A., Witherspoon K.T., Vives L., Patterson K.E. (2014). The contribution of de novo coding mutations to autism spectrum disorder. Nature.

[23] Wang T., Guo H., Xiong B., Stessman H.A.F., Wu H., Coe B.P., Turner T.N., Liu Y., Zhao W., Hoekzema K. (2016). De novo genic mutations among a Chinese autism spectrum disorder cohort. Nat. Commun..

[24] Stessman H.A.F., Xiong B., Coe B.P., Wang T., Hoekzema K., Fenckova M., Kvarnung M., Gerdts J., Trinh S., Cosemans N. (2017). Targeted sequencing identifies 91 neurodevelopmental-disorder risk genes with autism and developmental-disability biases. Nat. Genet..

[25] Talkowski M.E., Rosenfeld J.A., Blumenthal I., Pillalamarri V., Chiang C., Heilbut A., Ernst C., Hanscom C., Rossin E., Lindgren A.M. (2012). Sequencing chromosomal abnormalities reveals neurodevelopmental loci that confer risk across diagnostic boundaries. Cell.

[26] Yasin H., Gibson W.T., Langlois S., Stowe R.M., Tsang E.S., Lee L., Poon J., Tran G., Tyson C., Wong C.K. (2019). A distinct neurodevelopmental syndrome with intellectual disability, autism spectrum disorder, characteristic facies, and macrocephaly is caused by defects in CHD8. J. Hum. Genet..

[27] Ostrowski P.J., Zachariou A., Loveday C., Beleza-Meireles A., Bertoli M., Dean J., Douglas A.G.L., Ellis I., Foster A., Graham J.M. (2019). The CHD8 overgrowth syndrome: A detailed evaluation of an emerging overgrowth phenotype in 27 patients. Am. J. Med. Genet. C Semin. Med. Genet..

[28] An Y., Zhang L., Liu W., Jiang Y., Chen X., Lan X., Li G., Hang Q., Wang J., Gusella J.F. (2020). De novo variants in the Helicase-C domain of CHD8 are associated with severe phenotypes including autism, language disability and overgrowth. Hum. Genet..

[29] Wu H., Li H., Bai T., Han L., Ou J., Xun G., Zhang Y., Wang Y., Duan G., Zhao N. (2020). Phenotype-to-genotype approach reveals head-circumference-associated genes in an autism spectrum disorder cohort. Clin. Genet..

[30] Beighley J.S., Hudac C.M., Arnett A.B., Peterson J.L., Gerdts J., Wallace A.S., Mefford H.C., Hoekzema K., Turner T.N., O’Roak B.J. (2020). Clinical Phenotypes of Carriers of Mutations in *CHD8* or Its Conserved Target Genes. Biol. Psychiatry.

[31] Chenn A. (2008). Wnt/beta-catenin signaling in cerebral cortical development. Organogenesis.

[32] Sakamoto I., Kishida S., Fukui A., Kishida M., Yamamoto H., Hino S., Michiue T., Takada S., Asashima M., Kikuchi A. (2000). A novel beta-catenin-binding protein inhibits beta-catenin-dependent Tcf activation and axis formation. J. Biol. Chem..

[33] Nishiyama M., Nakayama K., Tsunematsu R., Tsukiyama T., Kikuchi A., Nakayama K.I. (2004). Early embryonic death in mice lacking the beta-catenin-binding protein Duplin. Mol. Cell. Biol..

[34] Nishiyama M., Oshikawa K., Tsukada Y., Nakagawa T., Iemura S., Natsume T., Fan Y., Kikuchi A., Skoultchi A.I., Nakayama K.I. (2009). CHD8 suppresses p53-mediated apoptosis through histone H1 recruitment during early embryogenesis. Nat. Cell Biol..

[35] Durak O., Gao F., Kaeser-Woo Y.J., Rueda R., Martorell A.J., Nott A., Liu C.Y., Watson L.A., Tsai L.-H. (2016). Chd8 mediates cortical neurogenesis via transcriptional regulation of cell cycle and Wnt signaling. Nat. Neurosci..

[36] Katayama Y., Nishiyama M., Shoji H., Ohkawa Y., Kawamura A., Sato T., Suyama M., Takumi T., Miyakawa T., Nakayama K.I. (2016). CHD8 haploinsufficiency results in autistic-like phenotypes in mice. Nature.

[37] Platt R.J., Zhou Y., Slaymaker I.M., Shetty A.S., Weisbach N.R., Kim J.-A., Sharma J., Desai M., Sood S., Kempton H.R. (2017). Chd8 mutation leads to autistic-like behaviors and impaired striatal circuits. Cell Rep..

[38] Gompers A.L., Su-Feher L., Ellegood J., Copping N.A., Riyadh M.A., Stradleigh T.W., Pride M.C., Schaffler M.D., Wade A.A., Catta-Preta R. (2017). Germline Chd8 haploinsufficiency alters brain development in mouse. Nat. Neurosci..

[39] Suetterlin P., Hurley S., Mohan C., Riegman K.L.H., Pagani M., Caruso A., Ellegood J., Galbusera A., Crespo-Enriquez I., Michetti C. (2018). Altered Neocortical Gene Expression, Brain Overgrowth and Functional Over-Connectivity in Chd8 Haploinsufficient Mice. Cereb. Cortex.

[40] Jung H., Park H., Choi Y., Kang H., Lee E., Kweon H., Roh J.D., Ellegood J., Choi W., Kang J. (2018). Sexually dimorphic behavior, neuronal activity, and gene expression in Chd8-mutant mice. Nat. Neurosci..

[41] Zhao C., Dong C., Frah M., Deng Y., Marie C., Zhang F., Xu L., Ma Z., Dong X., Lin Y. (2018). Dual Requirement of CHD8 for Chromatin Landscape Establishment and Histone Methyltransferase Recruitment to Promote CNS Myelination and Repair. Dev. Cell.

[42] Latcheva N.K., Delaney T.L., Viveiros J.M., Smith R.A., Bernard K.M., Harsin B., Marenda D.R., Liebl F.L.W. (2019). The CHD Protein, Kismet, is Important for the Recycling of Synaptic Vesicles during Endocytosis. Sci. Rep..

[43] Ellingford R.A., de Meritens E.R., Shaunak R., Naybour L., Basson M.A., Andreae L.C. (2020). Cell-type-specific synaptic imbalance and disrupted homeostatic plasticity in cortical circuits of ASD-associated *Chd8* haploinsufficient mice. bioRxiv.

[44] McDiarmid T.A., Belmadani M., Liang J., Meili F., Mathews E.A., Mullen G.P., Hendi A., Wong W.-R., Rand J.B., Mizumoto K. (2020). Systematic phenomics analysis of autism-associated genes reveals parallel networks underlying reversible impairments in habituation. Proc. Natl. Acad. Sci. USA.

[45] Rodríguez-Paredes M., Ceballos-Chávez M., Esteller M., García-Domínguez M., Reyes J.C. (2009). The chromatin remodeling factor CHD8 interacts with elongating RNA polymerase II and controls expression of the cyclin E2 gene. Nucleic Acids Res..

[46] Menon T., Yates J.A., Bochar D.A. (2010). Regulation of androgen-responsive transcription by the chromatin remodeling factor CHD8. Mol. Endocrinol..

[47] Subtil-Rodríguez A., Vázquez-Chávez E., Ceballos-Chávez M., Rodríguez-Paredes M., Martín-Subero J.I., Esteller M., Reyes J.C. (2014). The chromatin remodeller CHD8 is required for E2F-dependent transcription activation of S-phase genes. Nucleic Acids Res..

[48] Ceballos-Chávez M., Subtil-Rodríguez A., Giannopoulou E.G., Soronellas D., Vázquez-Chávez E., Vicent G.P., Elemento O., Beato M., Reyes J.C. (2015). The chromatin Remodeler CHD8 is required for activation of progesterone receptor-dependent enhancers. PLoS Genet..

[49] Hoffmann A., Zimmermann C.A., Spengler D. (2015). Molecular epigenetic switches in neurodevelopment in health and disease. Front. Behav. Neurosci..

[50] Hoffmann A., Sportelli V., Ziller M., Spengler D. (2017). Switch-Like Roles for Polycomb Proteins from Neurodevelopment to Neurodegeneration. Epigenomes.

[51] Elsabbagh M., Divan G., Koh Y.-J., Kim Y.S., Kauchali S., Marcín C., Montiel-Nava C., Patel V., Paula C.S., Wang C. (2012). Global prevalence of autism and other pervasive developmental disorders. Autism Res..

[52] Menger Y., Bettscheider M., Murgatroyd C., Spengler D. (2010). Sex differences in brain epigenetics. Epigenomics.

[53] Kandel E.R., Schwartz J.H., Jessel T.M., Siegelbaum S.A., Hudspeth A.J., Mack S. (2013). Principles of Neural Science.

[54] Belmonte M.K., Allen G., Beckel-Mitchener A., Boulanger L.M., Carper R.A., Webb S.J. (2004). Autism and abnormal development of brain connectivity. J. Neurosci..

[55] Picci G., Gotts S.J., Scherf K.S. (2016). A theoretical rut: Revisiting and critically evaluating the generalized under/over-connectivity hypothesis of autism. Dev. Sci..

[56] Bedford R., Pickles A., Lord C. (2016). Early gross motor skills predict the subsequent development of language in children with autism spectrum disorder. Autism Res..

[57] Merner N., Forgeot d’Arc B., Bell S.C., Maussion G., Peng H., Gauthier J., Crapper L., Hamdan F.F., Michaud J.L., Mottron L. (2016). A de novo frameshift mutation in chromodomain helicase DNA-binding domain 8 (CHD8): A case report and literature review. Am. J. Med. Genet. A.

[58] Werling D.M., Parikshak N.N., Geschwind D.H. (2016). Gene expression in human brain implicates sexually dimorphic pathways in autism spectrum disorders. Nat. Commun..

[59] Barak B., Feng G. (2016). Neurobiology of social behavior abnormalities in autism and Williams syndrome. Nat. Neurosci..

[60] Kim Y., Yang G.R., Pradhan K., Venkataraju K.U., Bota M., García Del Molino L.C., Fitzgerald G., Ram K., He M., Levine J.M. (2017). Brain-wide Maps Reveal Stereotyped Cell-Type-Based Cortical Architecture and Subcortical Sexual Dimorphism. Cell.

[61] Hardan A.Y., Fung L.K., Frazier T., Berquist S.W., Minshew N.J., Keshavan M.S., Stanley J.A. (2016). A proton spectroscopy study of white matter in children with autism. Prog. Neuropsychopharmacol. Biol. Psychiatry.

[62] Deoni S., Dean D., Joelson S., O’Regan J., Schneider N. (2018). Early nutrition influences developmental myelination and cognition in infants and young children. Neuroimage.

[63] Douzgou S., Liang H.W., Metcalfe K., Somarathi S., Tischkowitz M., Mohamed W., Kini U., McKee S., Yates L., Bertoli M. (2019). The clinical presentation caused by truncating CHD8 variants. Clin. Genet..

[64] Elbaz B., Popko B. (2019). Molecular Control of Oligodendrocyte Development. Trends Neurosci..

[65] Marie C., Clavairoly A., Frah M., Hmidan H., Yan J., Zhao C., Van Steenwinckel J., Daveau R., Zalc B., Hassan B. (2018). Oligodendrocyte precursor survival and differentiation requires chromatin remodeling by Chd7 and Chd8. Proc. Natl. Acad. Sci. USA.

[66] He D., Marie C., Zhao C., Kim B., Wang J., Deng Y., Clavairoly A., Frah M., Wang H., He X. (2016). Chd7 cooperates with Sox10 and regulates the onset of CNS myelination and remyelination. Nat. Neurosci..

[67] Batsukh T., Pieper L., Koszucka A.M., von Velsen N., Hoyer-Fender S., Elbracht M., Bergman J.E.H., Hoefsloot L.H., Pauli S. (2010). CHD8 interacts with CHD7, a protein which is mutated in CHARGE syndrome. Hum. Mol. Genet..

[68] Doherty G.J., McMahon H.T. (2009). Mechanisms of Endocytosis. Annu. Rev. Biochem..

[69] Nelson S.B., Valakh V. (2015). Excitatory/Inhibitory Balance and Circuit Homeostasis in Autism Spectrum Disorders. Neuron.

[70] Turrigiano G.G. (1999). Homeostatic plasticity in neuronal networks: The more things change, the more they stay the same. Trends Neurosci..

[71] Antoine M.W., Langberg T., Schnepel P., Feldman D.E. (2019). Increased Excitation-Inhibition Ratio Stabilizes Synapse and Circuit Excitability in Four Autism Mouse Models. Neuron.

[72] Tatavarty V., Torrado Pacheco A., Groves Kuhnle C., Lin H., Koundinya P., Miska N.J., Hengen K.B., Wagner F.F., Van Hooser S.D., Turrigiano G.G. (2020). Autism-Associated Shank3 Is Essential for Homeostatic Compensation in Rodent V1. Neuron.

[73] Carney D.S., Davies B.A., Horazdovsky B.F. (2006). Vps9 domain-containing proteins: Activators of Rab5 GTPases from yeast to neurons. Trends Cell Biol..

[74] Südhof T.C. (2017). Synaptic Neurexin Complexes: A Molecular Code for the Logic of Neural Circuits. Cell.

[75] McDiarmid T.A., Bernardos A.C., Rankin C.H. (2017). Habituation is altered in neuropsychiatric disorders-A comprehensive review with recommendations for experimental design and analysis. Neurosci. Biobehav. Rev..

[76] Villa C.E., Cheroni C., López-Tóbon A., Dotter C.P., Oliveira B., Sacco R., Yahya A.C., Morandell J., Gabriele M., Sommer C. (2020). *CHD8* haploinsufficiency alters the developmental trajectories of human excitatory and inhibitory neurons linking autism phenotypes with transient cellular defects. bioRxiv.

[77] Jin X., Simmons S.K., Guo A., Shetty A.S., Ko M., Nguyen L., Jokhi V., Robinson E., Oyler P., Curry N. (2020). In vivo Perturb-Seq reveals neuronal and glial abnormalities associated with autism risk genes. Science.

[78] Treutlein B., Camp J.G. (2020). Sequencing perturbed cortex development. Science.

[79] Gandal M.J., Leppa V., Won H., Parikshak N.N., Geschwind D.H. (2016). The road to precision psychiatry: Translating genetics into disease mechanisms. Nat. Neurosci..

[80] Tsankova N., Renthal W., Kumar A., Nestler E.J. (2007). Epigenetic regulation in psychiatric disorders. Nat. Rev. Neurosci..

[81] Ma D.K., Marchetto M.C., Guo J.U., Ming G., Gage F.H., Song H. (2010). Epigenetic choreographers of neurogenesis in the adult mammalian brain. Nat. Neurosci..

[82] Florio M., Borrell V., Huttner W.B. (2017). Human-specific genomic signatures of neocortical expansion. Curr. Opin. Neurobiol..

[83] Hoffmann A., Ziller M., Spengler D. (2019). Progress in iPSC-Based Modeling of Psychiatric Disorders. Int. J. Mol. Sci.

[84] Hoffmann A., Ziller M., Spengler D. (2020). Focus on Causality in ESC/iPSC-Based Modeling of Psychiatric Disorders. Cells.

